# CD23-negative atypical chronic lymphocytic leukemia with a novel three-way translocation t(4;10;13), unmutated: immunoglobulin heavy chain variable gene, and stereotyped subset #6 (case report)

**DOI:** 10.11604/pamj.2026.54.40.53226

**Published:** 2026-06-10

**Authors:** Rachida Guaouguaou, Amine Jebbor, Khalil Lotfi, Seloua Imane Ziani

**Affiliations:** 1 Laboratory of Research and Medical Analysis of the Royal Gendarmerie, Avenue Ibn Sina, Agdal, Rabat 10100, Morocco,; 2 Faculty of Medicine and Pharmacy, Mohammed V University, Rabat, Morocco,; 3 Mohammed V Military Teaching Hospital, Rabat, Morocco

**Keywords:** Chronic lymphocytic leukemia, CD23-negative Chronic lymphocytic leukemia, immunoglobulin heavy chain variable gene mutation, cytogenetics, case report

## Abstract

Chronic lymphocytic leukemia (CLL) is defined by the clonal expansion of mature B lymphocytes co-expressing CD19, CD5, and CD23. CD23 negativity is uncommon and creates real diagnostic difficulty, mainly because it overlaps with the mantle cell lymphoma (MCL) phenotype. We report a 55-year-old man who presented with massive lymphocytosis (595,000/mm^3^) and bilateral lymphadenopathy. Immunophenotyping showed a CD19+/CD5+/CD23- B-cell clone with a Matutes score of 4, establishing CLL diagnosis. Conventional karyotyping revealed a previously unreported three-way translocation t(4;10;13) (q32;q23;q14) alongside del(8)(q12q21) in 14 of 15 metaphases; FISH excluded TP53 deletion. Molecular analysis confirmed unmutated IGHV (100% germline homology) and stereotyped subset #6; two independent markers of poor prognosis that together signal constitutively active B-cell receptor signaling and antigen-driven disease progression. The patient sustained a traumatic brain injury shortly after diagnosis and died of refractory septic shock before CLL-directed therapy could begin. This case illustrates how adverse biological features can converge in the absence of TP53 disruption, and why full characterization, including immunophenotyping, metaphase cytogenetics, FISH, and molecular sequencing, is necessary to capture the true risk profile of the disease.

## Introduction

Chronic lymphocytic leukemia is the most common adult leukemia in Western countries. Its characteristic is the clonal accumulation, rather than rapid proliferation, of immunologically incompetent mature B lymphocytes in blood, marrow, and lymphoid tissues [1]. The disease is biologically heterogeneous: the same morphology can conceal vastly different molecular architectures, and those differences drive prognosis more than clinical stage alone [2]. CD23 is not simply a diagnostic marker. Its surface expression reflects B-cell activation through IL-4 signaling, and its presence is what separates CLL from MCL among CD5-positive proliferations [3]. When CD23 is absent, the differential cannot be resolved by phenotype alone; it requires mechanistic evidence, specifically, the absence of BCL-1 rearrangement and cyclin D1 overexpression [4]. Complex chromosomal rearrangements are a separate layer of risk. Unlike the recurrent FISH lesions; 13q deletion, trisomy 12, 11q deletion and 17p deletion, which each disrupt identifiable pathways, complex translocations signal genomic instability broadly and cluster disproportionately in unmutated IGHV cases [5,6]. We report a case that brings these elements together: CD23-negative CLL with a previously undescribed three-way translocation t(4;10;13), concurrent del(8q), 100% germline IGHV homology, and stereotyped subset #6. Each feature independently confers poor prognosis; their co-occurrence in one patient makes a compelling case for comprehensive biological workup beyond standard FISH panels [7].

## Patient and observation

**Patient information:** a 55-year-old man with no significant prior medical history was referred to the Ear, Nose, and Throat (ENT) department for progressive, painless right cervical swelling. There was no relevant family history of hematologic malignancy. Ultrasound confirmed multiple bilateral cervical and axillary lymphadenopathies. A pre-operative biological workup was requested in preparation for a cervical lymph node biopsy, without prior suspicion of a lymphoproliferative disorder. It was during the analysis of this routine workup that a striking lymphocytosis was identified on the peripheral blood smear, prompting the clinical biologist to independently initiate flow cytometry immunophenotyping, which led incidentally to the diagnosis of CLL prior to any surgical procedure.

**Clinical findings:** examination revealed firm, non-inflammatory right cervical lymphadenopathy (Figure 1). The complete blood count showed massive leukocytosis at 595,000/mm^3^, with an absolute lymphocyte count of 583,100/mm^3^. Peripheral blood smear was dominated by small, mature-appearing lymphocytes with condensed chromatin and scant cytoplasm (Figure 2). Flow cytometry identified a monoclonal B-cell population: CD19+, CD5+, CD20+, CD23-, FMC7-, CD79b-, with weak kappa light chain restriction. Matutes score: 4/5.

**Figure 1:**
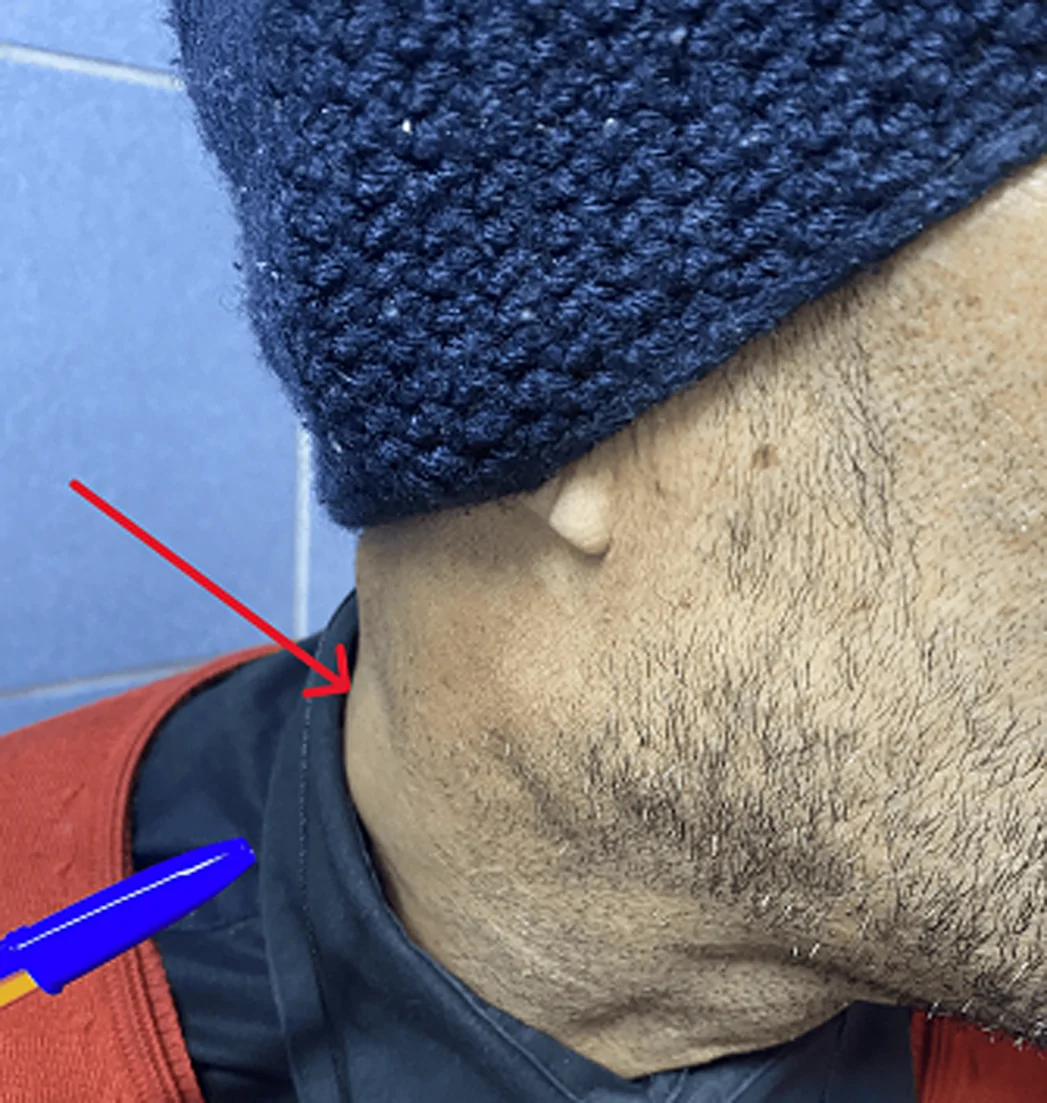
clinical image showing a firm, non-inflammatory right cervical lymphadenopathy (indicated by arrow) at initial presentation

**Figure 2:**
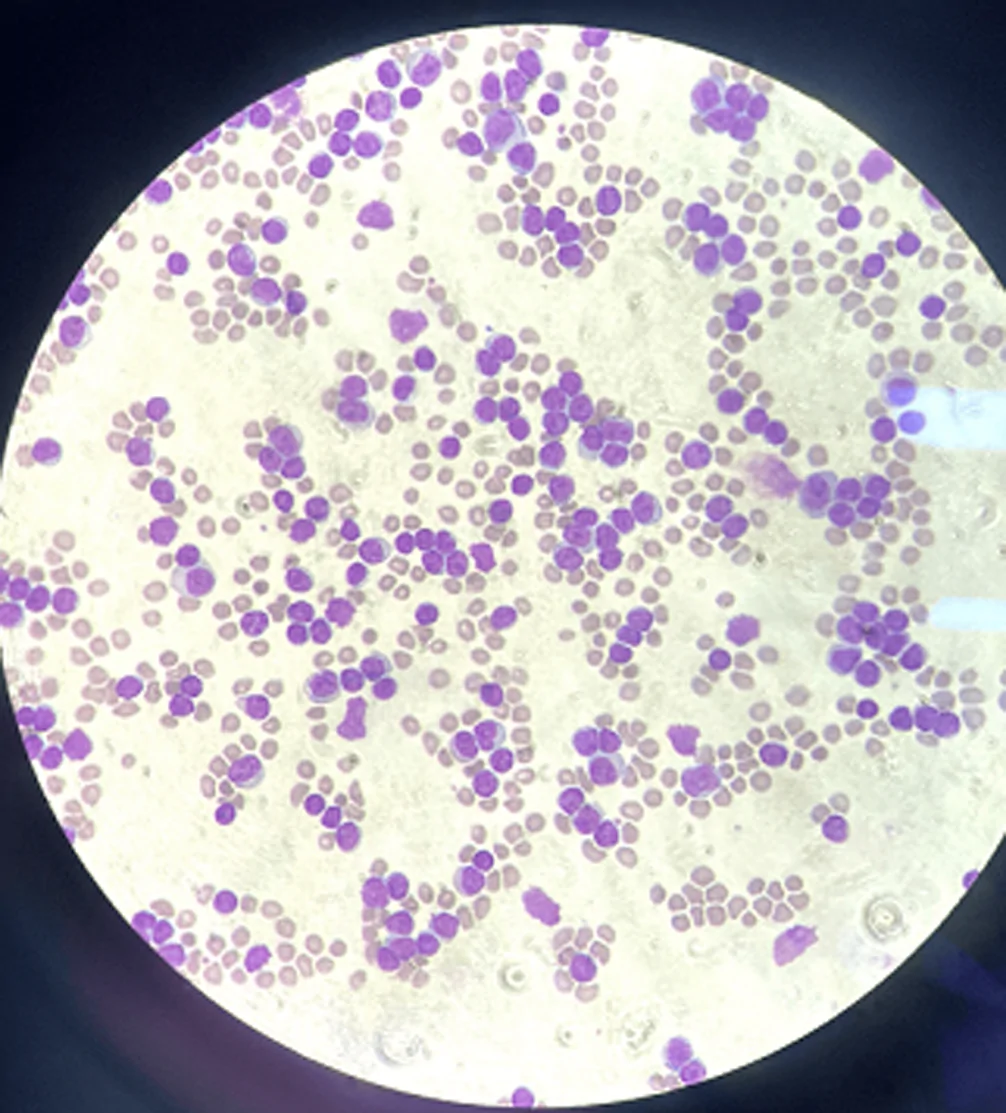
peripheral blood smear (May-Grünwald-Giemsa, x50); massive lymphocytosis composed of small, mature lymphocytes with condensed chromatin and scant cytoplasm; white blood cells 595,000/mm^3^

**Timeline:** the chronology of this case is summarized in Table 1. The patient was referred to the ENT department for progressive right cervical swelling. Ultrasound revealed bilateral lymphadenopathies, and a cervical lymph node biopsy was planned. A routine pre-operative biological workup was requested in this context, without any suspicion of a hematologic malignancy. On reviewing the complete blood count, the clinical biologist identified a striking lymphocytosis on the peripheral blood smear and performed flow cytometric immunophenotyping on their own initiative, leading to the incidental diagnosis of CLL. The planned biopsy was consequently cancelled. The patient was directly contacted by the biological team, and the ENT physician was notified and advised to orient the patient toward clinical hematology for rapid management. Cytogenetic and molecular analyses followed, revealing a complex karyotype and an unfavorable molecular profile. Shortly after completion of the workup, the patient suffered a traumatic brain injury and was admitted to the ICU, where he died of refractory septic shock before any CLL-directed therapy could be initiated.

**Table 1:** timeline of clinical events from initial presentation to death

Period	Event
Weeks before the consultation	Patient notices progressive, painless right cervical swelling; attributed to a benign cause
Day 0 - ENT referral	Referred for cervical lymphadenopathy; ultrasound confirms bilateral cervical and axillary lymphadenopathies
Day 0 - laboratory (biologist's initiative)	CBC reveals leukocytosis (595,000/mm^3^); peripheral smear shows mature lymphocytosis; flow cytometry performed on biologist's own initiative identifies CD19+/CD5+/CD23- monoclonal B-cell population; CLL diagnosis established; patient contacted directly; ENT physician notified; patient oriented toward clinical hematology
Days 1-7	Conventional cytogenetics: novel three-way translocation t(4;10;13)(q32;q23;q14) + del(8)(q12q21) in 14/15 metaphases; FISH: TP53 intact
Days 7-14	Molecular analysis: IGHV unmutated (100% germline homology); stereotyped subset #6 identified
Post-workup	Patient suffers traumatic brain injury; admitted to ICU
ICU course	Refractory septic shock; death before any CLL-directed therapy was initiated

ENT: ear, nose and throat; ICU: intensive care unit; CBC: complete-blood-count; CLL: chronic lymphocytic leukemia; IGHV: immunoglobulin heavy chain variable gene; FISH: fluorescence in situ hybridization

**Diagnostic assessment:** conventional cytogenetics was performed on peripheral blood after 72-hour B-cell mitogen culture. Fifteen metaphases were analyzed; 14 showed a pseudodiploid karyotype: *46,XY,t(4;10;13)(q32;q23;q14),del(8)(q12q21)*[14]*/46,XY*[1] FISH using TP53/CEN17 probes found no TP53 deletion or gain in 23 metaphases and 200 interphase nuclei. Multiplex PCR and sequencing of IGHV demonstrated 100% germline homology (unmutated by ERIC criteria, cutoff ≥98%), with subset analysis identifying stereotyped subset #6.

**Diagnosis:** CD23-negative atypical CLL with complex cytogenetics and unfavorable molecular profile. MCL was excluded by the absence of BCL-1 rearrangement and the immunophenotypic convergence supporting CLL (Matutes 4/5, FMC7-, CD79b-).

**Therapeutic interventions and follow-up:** no CLL-directed therapy was initiated. Following completion of the diagnostic workup, the patient sustained a traumatic brain injury and was admitted to the intensive care unit. Despite maximal supportive care, he died of refractory septic shock. No adverse events related to CLL therapy occurred, as no treatment had yet been administered. The outcome in this case was therefore determined entirely by an intercurrent event, independently of the underlying tumour biology. Regular follow-up contact was maintained between the patient and the biological team throughout the diagnostic process. The patient´s death was learned of during a follow-up telephone call, during which his daughter reported that he had passed away fifteen days earlier.

**Patient perspective:** the diagnosis of CLL in this patient originated from a biologist´s initiative rather than a formal clinical request, and a direct relationship was established with the patient throughout the diagnostic workup. He willingly shared his imaging results and clinical history, remained engaged and cooperative at each step, and expressed a clear willingness to begin treatment once the full workup was complete. He promised to keep the biological team informed of his clinical course. Several weeks passed without news. When contacted for follow-up, it was his daughter who answered, informing us that he had passed away fifteen days earlier, before any CLL-directed therapy could be initiated. His family subsequently provided written informed consent for the publication of this case report, expressing the hope that his experience might contribute to the understanding of atypical CLL presentations and benefit future patients.

**Informed consent:** written informed consent was obtained from the patient´s family for publication of this case report and accompanying images.

## Discussion

The diagnostic problem here was mechanistically defined. CD23 is regulated through IL-4 signaling and marks the post-germinal centre B-cell lineage typical of CLL. Its absence does not exclude CLL but it narrows the diagnostic space, because MCL shares the CD5+/CD23- phenotype. What distinguishes the two is not phenotype but mechanism: MCL is driven by t(11;14)-mediated cyclin D1 overexpression, which was absent here. The Matutes score of 4, the absence of FMC7 and CD79b, and the lack of BCL-1 rearrangement collectively resolve the differential without ambiguity [3,4,8]. The absence of TP53 deletion by FISH has important therapeutic implications. Del(17p) causes marked resistance to chemoimmunotherapy by disrupting the DNA damage response; its absence preserves a broader therapeutic window and changes the expected treatment trajectory. That window was never used here, but its existence matters for understanding the case [5]. The cytogenetic picture is the most unusual feature. A three-way translocation t(4;10;13)(q32;q23;q14) with concurrent del(8)(q12q21) in 14 of 15 metaphases represents a clonal event with no prior report in CLL. The specific breakpoints implicate 4q32, 10q23 (the PTEN locus), and 13q14 (the miR-15a/16-1 locus), though functional consequences at each site require further study. More broadly, complex karyotypes in CLL; defined as three or more chromosomal abnormalities, independently shorten time to first treatment and compress overall survival, regardless of TP53 status. The mechanism is likely accelerated acquisition of secondary mutations driven by genomic instability [6,9].

IGHV mutation status adds a second, independent risk signal. A 100% germline homology means the CLL clone derives from a B cell that never underwent somatic hypermutation in the germinal center. Functionally, this is associated with chronic active B-cell receptor signaling that drives pro-survival signaling through Bruton's tyrosine kinase (BTK) and PI3Kδ. This is why unmutated IGHV predicts shorter treatment-free survival and forms a core component of the CLL-International Prognostic Index (CLL-IPI), and why BTK inhibitors have a clear mechanistic rationale in this setting [7,10]. Subset #6 (IGHV3-21/IGLV3-21) adds further complexity. This stereotyped BCR configuration drives aggressive disease independent of IGHV mutation status; a finding that breaks the binary mutated/unmutated risk framework and points toward antigen-driven clonal selection as a distinct pathological mechanism [7]. Both features acting together in one patient are not simply additive; they reflect convergent evidence of a high-risk tumour biology (Table 2) [11-15].

**Table 2:** summary of reported cases of atypical CD23-negative CLL with complex cytogenetics and/or unfavorable molecular profile

Authors/year	Sex	Age	CD23	Cytogenetics	IGHV status	Matutes	Treatment/outcome
Di Raimondo *et al*. 2002	M/F	Med. 62	Negative	Various; del(13q), trisomy 12 predominant	NR	3-4	CD23− CLL = 6% of CD5+/CD19+ cases; more aggressive vs CD23+ CLL
Lima *et al*. 2002	M	Elderly	Negative	t(11;14); bcl-1 excluded	NR	4	Tonsillar mass; CLL confirmed after BCL-1 exclusion; conservative management
Cennamo *et al*. 2023	M	63	Positive	Complex; t(6;11) unbalanced; del(6q), del(11q)/ATM loss	Unmutated	4	AIHA; rituximab then acalabrutinib; partial response
Stamatopoulos *et al*. 2007	M/F	Med. 58	Variable	Complex (≥3 aberrations); del(13q), del(11q), del(17p), translocations	Predominantly unmutated	3-4	Complex karyotype is independently associated with shorter TTFT and inferior OS
Davi *et al*. 2020	M/F	Variable	Variable	Subset #6: del(13q), del(11q) frequent	Mixed	3-5	Subset #6 associated with shorter TTFT regardless of IGHV status
Present case/2025	M	55	Negative	t(4;10;13)(q32;q23;q14), del(8)(q12q21); TP53 intact by FISH	Unmutated (100%); Subset #6	4	Died of septic shock after TBI; no CLL therapy initiated

M: male; F: female; CLL: chronic lymphocytic leukemia; MCL: mantle cell lymphoma; IGHV: immunoglobulin heavy chain variable gene; FISH: fluorescence in situ hybridization; TTFT: time to first treatment; OS: overall survival; NR: not reported; TBI: traumatic brain injury; AIHA: autoimmune haemolytic anaemia

What this case makes plain is that high-risk CLL does not require del(17p) as its foundation. Atypical immunophenotype, structural chromosomal complexity, unmutated IGHV, and high-risk BCR stereotypy can together define a disease as aggressive as any TP53-deleted case. Risk stratification that stops at FISH will systematically miss this. The ERIC guidelines are explicit: IGHV sequencing and subset assignment are constitutive elements of the diagnostic workup, not optional additions [2,7]. This case also illustrates a dimension of laboratory medicine that is rarely documented in case reports: the proactive role of the clinical biologist. The biological workup was requested solely in preparation for a cervical lymph node biopsy, with no prior suspicion of a hematologic malignancy. Upon identifying a striking lymphocytosis on the peripheral blood smear, flow cytometry was performed on the biologist's own initiative, leading incidentally to the diagnosis of CLL and rendering the planned biopsy unnecessary. The ENT physician was immediately notified and the patient oriented toward clinical hematology. This case is a reminder that the biologist's role is not limited to answering clinicians' requests - it can be decisive in early diagnosis, sparing patients unnecessary procedures, and ensuring timely specialist referral.

## Conclusion

This case combines an unusual set of adverse features in a single patient: CD23 negativity, a novel three-way translocation t(4;10;13) with del(8q), unmutated IGHV at 100% germline homology, and stereotyped subset #6. No single finding would be exceptional on its own. Together, they reflect the biological heterogeneity that makes CLL diagnostically demanding and prognostically unpredictable. The patient died from septic shock before any CLL-directed therapy could be initiated. This highlights how comorbid illness and intercurrent events may influence outcomes independently of the underlying tumour biology. Comprehensive characterization integrating phenotype, metaphase cytogenetics, FISH, and molecular sequencing remains the minimum standard the disease requires.
